# Spatio-temporal classification for polyp diagnosis

**DOI:** 10.1364/BOE.473446

**Published:** 2023-01-04

**Authors:** Juana González-Bueno Puyal, Patrick Brandao, Omer F. Ahmad, Kanwal K. Bhatia, Daniel Toth, Rawen Kader, Laurence Lovat, Peter Mountney, Danail Stoyanov

**Affiliations:** 1 Wellcome/EPSRC Centre for Interventional and Surgical Sciences (WEISS), University College London, London W1W 7TY, UK; 2 Odin Vision, London W1W 7TY, UK

## Abstract

Colonoscopy remains the gold standard investigation for colorectal cancer screening as it offers the opportunity to both detect and resect pre-cancerous polyps. Computer-aided polyp characterisation can determine which polyps need polypectomy and recent deep learning-based approaches have shown promising results as clinical decision support tools. Yet polyp appearance during a procedure can vary, making automatic predictions unstable. In this paper, we investigate the use of spatio-temporal information to improve the performance of lesions classification as adenoma or non-adenoma. Two methods are implemented showing an increase in performance and robustness during extensive experiments both on internal and openly available benchmark datasets.

## 1. Introduction

Colorectal cancer is the third most prevalent cancer worldwide and early detection and treatment can significantly improve the patient’s prognosis [1]. During colonoscopies the bowel is inspected, and diagnosis and treatment of pre-cancerous polyps is carried out [2]. Differentiating polyp types intra-operatively rather than relying on histology post-procedure can potentially minimise unnecessary interventions for harmless polyps, saving time and costs. Such strategies are already advised by the American Society for Gastrointestinal Endoscopy (ASGE) to avoid unnecessary histopathological analysis of diminutive ( ≤ 5mm) adenomatous lesions and to leave in situ hyperplastic polyps in the rectum or sigmoid [3], which has be shown to save significant healthcare costs [4]. Direct optical diagnosis of polyps can be attempted using chromoendoscopic image modalities such as narrow-band imaging (NBI) or Blue Laser Imaging (BLI) [5], and validated classification systems such as the NBI International Colorectal Endoscopic (NICE) classification [6]. However, this is challenging and performance varies significantly between novice and expert endoscopists [7–9].

Computer-aided diagnostic (CADx) systems can be used to augment optical diagnosis and differentiate between adenomatous, hyperplastic or sessile serrated polyps in the colon. Polyp classification methods have been widely investigated, focusing on classifying hyperplastic against adenomatous polyps [10–13] or on adenoma against non-adenoma classification (hyperplastic and sessile serrated lesions) [10,12,14–17]. In preclinical studies, it has been shown that such CADx systems can be used as a decision support tool, allowing novice endoscopists to reach near-expert levels of accuracy [13].

Technically, most recent CADx approaches are based on deep learning with a range of architectures being reported [18] including semi-supervised learning [19] and the fusion of different input modalities [20]. Endoscopes providing magnified chromoendoscopy enhance the mucosal patterns and improve optical diagnosis. Some studies focus on the use this type of data for classification of colorectal polyps with promising results [21–24]. However, this type of endoscope is not widely available and CADx systems that can be used on non-magnified data are extremely useful.

**Fig. 1.**
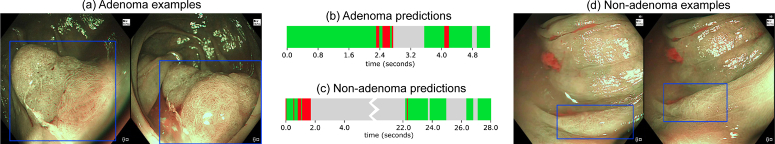
Examples of polyp appearance variation (with expert polyp boxes in blue) for (a) an adenoma and (d) non-adenoma polyp. The timelines (middle) show example predictions on the adenoma video sequence (b) and non-adenoma sequence (c) - green, red and grey denote correct and incorrect predictions and non-annotated frames, respectively.

Machine learning techniques rely on data, hence public polyp histopathology datasets have started becoming available [25–27]. Polyp datasets are curated and usually contain good-quality images with clear views of the polyp. These have been used to train and evaluate CADx models with very promising results based on different combinations of colour, texture and shape features [10,28–30]. However, such studies only train and test on selected high-quality frames, which does not demonstrate their generalisation capabilities to operate on real-time videos. One of the problems associated with clinical practice videos is that obtained views of the same polyp can vary greatly due to different camera orientations, changes in lighting, mucosa deformability, presence of artifacts and blurriness, etc. For applied clinical use, CADx systems need to be stable to such observational differences (see Fig. 1) and present consistent predictions for the same lesion. It is therefore important to evaluate the consistency, as well as to report results on a per-frame and per-polyp basis.

In practice, endoscopists use both spatial and temporal information when detecting and diagnosing polyps, where observing the polyp over consecutive frames in a video aids the task. The use of spatio-temporal information has been shown to improve other interventional applications, such as surgical phase recognition [31], polyp size estimation [32] or polyp detection [33–35]. In this study, we focus on incorporating such spatio-temporal information within polyp diagnosis CADx for the first time. We show how two different methods to incorporate temporal information for adenoma and non-adenoma classification can be implemented and the improvement over single shot classification that they can achieve. Long-short Term Memory (LSTM) networks continue to stand as one of the preferred ways to combine temporal information in medical videos [36–38]. Besides their high performance, LTSM modules are lighter than 3D architectures, which reduces overfitting when handling few videos. For these reasons, a method incorporating an LSTM module was used as one of the spatio-temporal methods, and was compared to simple but powerful temporal combinations of the predictions inspired by post-processing techniques in ensembling.

The proposed solutions were extensively evaluated, both on internal data using cross-validation and external data to evaluate generalisability. An in-depth evaluation of performance was carried out, reporting standard metrics as well as polyp accuracy in order to evaluate the consistency of predictions. Polyp classification needs to be carried out per lesion. To overcome the fact that several polyps can be in view simultaneously in a video frame, the classification is applied only to the region of the image containing the polyp. The spatio-temporal methods were tested recreating a clinical environment workflow using them in combination with a polyp detection model. This highlighted the benefits temporal methods bring in this setup. Finally, the polyp diagnosis methods were quantified in terms of the quality of the polyp location to evaluate the classification robustness.

## 2. Methods

**Fig. 2.**
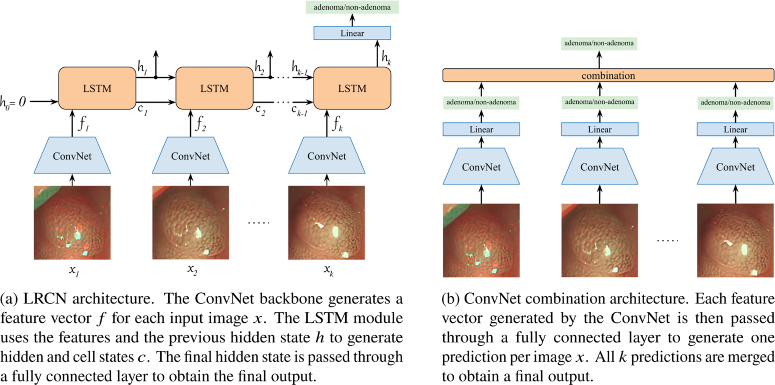
Architectures of the proposed spatio-temporal methods for adenoma/non-adenoma video clip classification.

Two spatio-temporal methods were implemented for adenoma/non-adenoma video clip classification, namely a Long-Term Recurrent Convolutional Network (LRCN) [39] and Convolutional Network (ConvNet) predictions combination. The LRCN 2D+t (frame-based approach with time) model was implemented to classify short video clips. In the second method, each video was decomposed into frames and each frame was first classified with a standard ConvNet, followed by combining the outputs to generate a final prediction. Several combination methods traditionally used for ensembles were explored, namely soft averaging, plurality vote and extreme vote. Figure 2 presents the networks’ architectures.

The models were trained for a maximum of 20 epochs, SGD as the optimizer with a learning rate of 0.0001. Cross-entropy loss was used using balanced class weights to assign each class weights inversely proportional to their respective frequencies. The overall balance of adenoma/non-adenoma boxes was 76%/24%, but it differed on each fold. Batch sizes were selected based on available GPU memory. The code was implemented on Pytorch 1.6 on Ubuntu 18.04.4 LTS with a GeForce RTX 2080 GPU.

Both methods were developed with a shared backbone, a Resnet50 ConvNet [40], in order to allow for comparison. The backbone was additionally used as a baseline for ablation studies analysis. Moreover, the explored architectures were studied following a full workflow setup where a polyp detection model predicted the location of the polyp in each image, then used for the classification task. In this section, the development of the methods is described, along with the data handling.

### 2.1 Datasets

A dataset of colonoscopic videos was used for our experiments. The videos were collected at University College Hospital in London between 2018 and 2021 (project ID 236056). All adenomatous polyps (tubular adenoma, villous adenoma, tubulovillous adenoma) and serrated polyps (hyperplastic, traditional serrated adenoma, sessile serrated lesion) were included. All other polyps were excluded. Videos were collected using Olympus 260 and Olympus 290 endoscopes (Olympus Lucera) and annotated by expert endoscopists to include a bounding box around visible polyps. Polyp-related image quality labels were also added, deeming the image as high-quality if the polyp(s) was discernible. Histology results were adopted for adenoma and non-adenoma ground-truth labels. Table 1 includes further information about the data. Only NBI frames containing annotated polyp boxes were considered, including NBI-Near Focus frames.

**Table 1.** Description of the internal and external (Piccolo Dataset [26]) datasets.

	Internal Dataset	External Dataset
Patients	215	40
**Lesions**	**419**	**76**
Diminutive polyps ( ≤ 5mm)	54.56%	27.63%
Histology (adenoma/non-adenoma)	59.35%/40.65%	65.79%/22.37%
**Frames registered**	**1,821,285**	**3,433**
NBI	11.76%	37.93%
NBI-Near Focus	9.18%	0%
Annotated polyp frames	7.04%	100.00%
High-quality	6.75%	-

Additionally, the Piccolo Dataset was used as an external testing set. It is a publicly available dataset that comprises 3433 manually annotated images (2131 white-light images 1302 narrow-band images), originated from 76 lesions from 40 patients using Olympus endoscopes (CF-H190L and CF-HQ190L). Low quality and uninformative frames were removed, and the videos were sampled every 25 frames. Each lesion has an associated histology as adenoma, hyperplasia or adenocarcinoma as well as a binary mask with the location of the polyp [26]. We considered hyperplastic polyps as non-adenoma, and excluded adenocarcinomas. Only NBI sequences were used in this study.

### 2.2 LRCN

A LRCN architecture was used to classify video clips as adenoma or non-adenoma. This architecture was selected because of its success on time-series tasks, and due to its ability to learn disentangled spatial and temporal representations. Other 3D models such as C3D extract spatio-temporal features, which can be useful for tasks such as action recognition where the objects movement is part of the action. However, in the case of polyp diagnosis where the video is egocentric, the motion of the camera does not determine the type of polyp. The LRCN architecture combined a deep visual feature extraction (such as a ConvNet) with a Long-Short Term Memory (LSTM) module to collate temporal dynamics for sequential data tasks [39].

In the current implementation, a Resnet50 backbone [40] was used as the deep encoder to extract a spatial feature representation. Its final fully connected layer was removed, the backbone generating a feature vector with 2048 elements per input frame. The ConvNet backbone was followed by a LSTM module, which was composed of a single layer with 100 hidden units. The size was chosen experimentally in order to balance performance against overfitting. A many-to-one structure was implemented, where for each clip input composed by k frames we used the output $h_{k}$ from the last frame iteration, as it encompassed temporal information from all previous frames in the clip. The 100 output features were finally passed through a fully connected layer to obtain two output classes, namely adenoma and non-adenoma. Figure 2(a) illustrates the architecture of our LRCN network.

The backbone was pretrained on the available frames and its weights frozen when training the LRCN, as this setup showed a small experimental improvement when compared to end-to-end training. The learning rate was reduced on plateau by a factor of 0.1 with a patience of four epochs. The overall inference speed was 72.21 frames/second on our GPU.

### 2.3 ConvNet predictions combination

The second spatio-temporal method utilised consisted on aggregating the ConvNet outputs. In this 2-step method, each visual input $x_{i}$ (a frame from the input video clip) was first passed through a Resnet50 ConvNet for spatial encoding to produce a continuous prediction p(y|x)∈[0,1] . For this first step other Resnet architectures were experimentally explored, but larger networks were found to overfit with the amount of data available. The network was pretrained from ImageNet weights. For all experiments, the Resnet50 was trained with a batch size of 64.

A second step was used to incorporate temporal information. Several methods were explored for this phase, namely soft averaging, plurality vote and extreme vote. In soft averaging, softmax outputs obtained from all frames in a clip of length k were averaged to obtained a temporally weighted output z for each clip, as described in Eq. (1).

(1) $$z=\frac{1}{k}\cdot \sum_{i=1}^{k}p(y|x_{i})$$

where $p(y|x_{i})$ corresponds to the probability prediction from the ConvNet after softmax for an input frame $x_{i}$ .

The plurality vote was obtained by thresholding predictions from each of the k frames and selecting the class label with the most votes. This generated binary predictions instead of continuous outputs. In the case of extreme voting, the frame output with the highest or lowest prediction was selected as the final prediction, as shown in Eq. (2).

(2) $$z=\max_{\forall i\in k}|p(y|x_{i})-0.5|$$

In the case of soft averaging and extreme voting, the final prediction z was finally thresholded with a value of 0.5 to obtain a final output y (Eq. (3)). The overall inference speed was 97.20 frames/second on our GPU.

(3) $$y=\left\{ \begin{array}{ll} 0 & \text{if}z\leq T\\ 1 & \text{if}z>T \end{array}\right.$$

where T is the selected threshold T=0.5 .

### 2.4 Data processing

A clip was defined as a set of k consecutive frames. Clips were extracted from the colonoscopic videos in a sliding window fashion with a stride of one to maximize the number of clips. For the internal dataset, frozen video sequences were excluded to ensure variation within the clips. Only clips where ≥ 50% of the frames were labelled as high-quality were included to simulate the clinical setup, where the endoscopist performs visual diagnosis once a good view of the polyp is obtained and the polyp features are visible. The ≥ 50% threshold was selected to ensure a balance between sufficient image quality and the amount of discarded data. In the Piccolo dataset consecutive frames were not available as the videos are sampled every 25 frames, so clips were composed of non-consecutive, ordered frames, and no clips were discarded due to image quality.

**Fig. 3.**
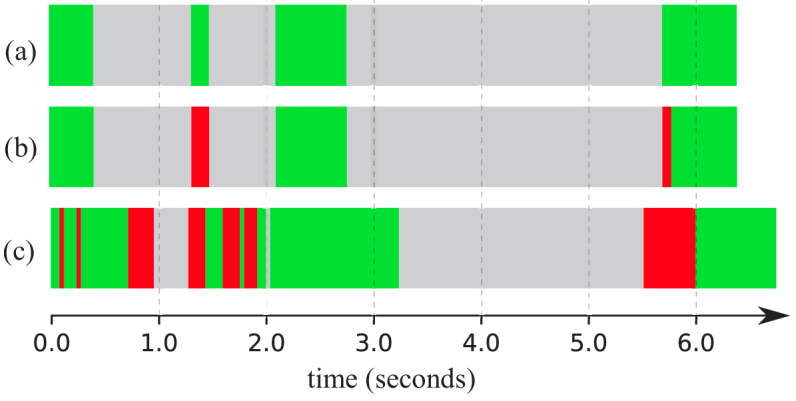
Prediction timelines for the same polyp sequence with (a) LRCN, (b) ConvNet averaging and (c) ConvNet - green, red and grey denote correct and incorrect predictions and non-annotated frames, respectively. Note: the spatio-temporal methods present shorter timelines as the last k−1=14 samples (0.6 seconds) did not have enough following frames to create a clip.

The LRCN model was trained with each clip as an input sample, whereas the ConvNet was trained with all the frames included within the LRCN clips, ensuring the same frames were utilised for both methods, although less samples were used for LRCN. After excluding white light sequences, low-quality clips and lesions with less than k frames, the baseline model was trained with a total of 27,087 frames from 197 polyps from 89 colonoscopy procedures, for k=15 . As a note, Fig. 3 shows an example of how clips were discarded when they contained non-annotated frames, reducing the amount of available samples.

Random sampling of 5000 frames was performed on each epoch, re-sampling each time, to minimise overfitting [41]. Data augmentation was applied in such a way as to guarantee identical augmentations within clips. The augmentation operations consisted of random affine transformations (rotation, translation and scaling) and random colour transformations (brightness, contrast and saturation). Finally, the images were preprocessed by cropping around the polyp boxes annotated by experts, followed by resizing the images to 224 by 224 pixels and an intensity normalization step. Only the polyp area was used as an input to the networks, discarding the remainder of the image. This ensures that the adenoma classification model can be used in a clinical setting, where more than one polyp can be present in a frame.

All models were trained with 5-fold patient cross-validation. For all experiments, the same folds were respected, to ensure a fair comparison between models. For each fold, each patient’s video was used for either training or testing following an 80-20% split, avoiding any data contamination. The patient splits were generated optimizing the distribution balance between the training and testing sets in terms of the number of NBI polyp frames, the number of different lesions, the polyp size (in pixels), the polyp types and the quality of the images.

### 2.5 Detection and classification pipeline setup

In a clinical setup, the locations of the polyps in each colonoscopy frame would not be provided by experts, but by a computer-assisted detection (CAD) model. It was paramount to implement such a pipeline consisting of a polyp detection model followed by a polyp classification model, in order to assess the full polyp classification workflow.

A polyp segmentation model, an FCN-Resnet101, was trained on our internal dataset using the same 5-fold cross-validation splits used for the previous experiments. A new set of predicted bounding boxes was obtained on frames from each of the 5-fold testing sets using the polyp detection network results as follows: (i) frames containing multiple polyps were discarded; (ii) if only one region was predicted as a polyp, the detected region was circumscribed by a rectangular bounding box; (iii) if multiple regions were detected (when false positives occurred), they were enclosed as a single prediction in the same bounding box; (iv) if no polyp was detected, the frame was discarded as no box could be used to crop the image.

## 3. Experimental results

### 3.1 Evaluation metrics

Traditional metrics were used for the evaluation of the methods, namely accuracy, sensitivity and specificity (always using a threshold of 50%) as well as area under the curve (AUC). These metrics were computed using all boxes from all evaluated frames. When testing on internal data, the results from all folds were aggregated together and the metrics were computed on the entire dataset. Additionally, we introduced polyp accuracy. It quantifies the percentage of correctly predicted frames in a polyp, averaged across all polyps. In Tables 2, 3 and 4, polyp accuracy is given as the mean of all polyp accuracies, with a 95% confidence interval (CI). Polyp accuracy allows knowing if, on average, the polyps have a high or low per-frame accuracy. Because high per-frame accuracy for a polyp means high consistency in the predictions, this metric gives an indication of the robustness of the models to temporal differences.

### 3.2 Baseline performance

A Resnet50 was trained as our baseline ConvNet. Other architectures were explored for polyp classification, but Resnet architectures showed the best results empirically. Different model sizes were explored, however Resnet50 gave a good balance between performance, training time and generalisability. In order to allow for comparison with other methods, the frames used for the baseline were the same frames used for the temporal experiments, containing images from a 15 frames clip extraction using 5-fold cross-validation, as detailed in Section 2.4.

**Table 2.** Polyp diagnosis cross-validation results the internal dataset.

Method	Clip size	Accuracy(%)	Sensitivity(%)	Specificity(%)	AUC(%)	Polyp accuracy(%) [95% CI]
ConvNet	N/A	81.67	83.91	76.21	88.61	77.13 [73.01, 81.25]
LRCN	15	**86.02**	**89.62**	77.08	**92.60**	**80.34 [76.00, 84.68]**
ConvNet soft averaging	15	84.64	85.70	82.03	91.93	79.20 [74.51, 83.90]
ConvNet plurality vote	15	84.91	85.85	**82.56**	84.21	79.49 [74.83, 84.16]
ConvNet extreme vote	15	83.15	83.56	82.13	90.86	77.76 [73.03, 82.48]

As it can be observed in Table 2, the Resnet50 model achieves an 88.61% AUC, with per-frame sensitivity surpassing 80% in our internal dataset. It is crucial to evaluate the per-polyp accuracy, as it reflects the distribution of correct/incorrect predictions throughout the polyps. In this case, a drop in accuracy occurs when evaluating per polyp, due to the fact that longer polyp videos in this dataset perform better than shorter videos. On average, the baseline model will correctly predict 77.13% [95% CI: (73.01, 81.25)] of the frames for a polyp. In practical terms, around a quarter of the predictions will fail for each polyp, showing a lack of consistency when predicting on different frames of the same lesion. In clinical practice this poses problems in terms of trust towards the CADx model and reduces the usability of the system.

### 3.3 Effect of temporal methods on polyp diagnosis

The weights from the Resnet50 ConvNet were used to initialise the LRCN backbone. Additionally, the ConvNet baseline predictions were combined for each 15-frame clip in the test set to obtain per-clip results. It is important to note that, even though all methods were tested on the same frames, the baseline was evaluated per frame, whereas the temporal methods were evaluated on a per-clip basis. As it can be observed in Table 2, all methods incorporating temporal information surpassed the ConvNet baseline performance when evaluating with traditional metrics, with up to a ∼ 3% increase in the area under the curve (AUC) with LRCN. Regarding ConvNet combination methods, extreme voting presents a lower performance than soft averaging and plurality vote across all metrics. Soft averaging and plurality perform similarly, but soft averaging was found preferable as the threshold can be calibrated for this method. In further experiments, soft averaging was selected as the optimal ConvNet combination method. In turn, ConvNet soft averaging and LRCN show similar results with a different balance between sensitivity and specificity but similar AUC.

**Fig. 4.**
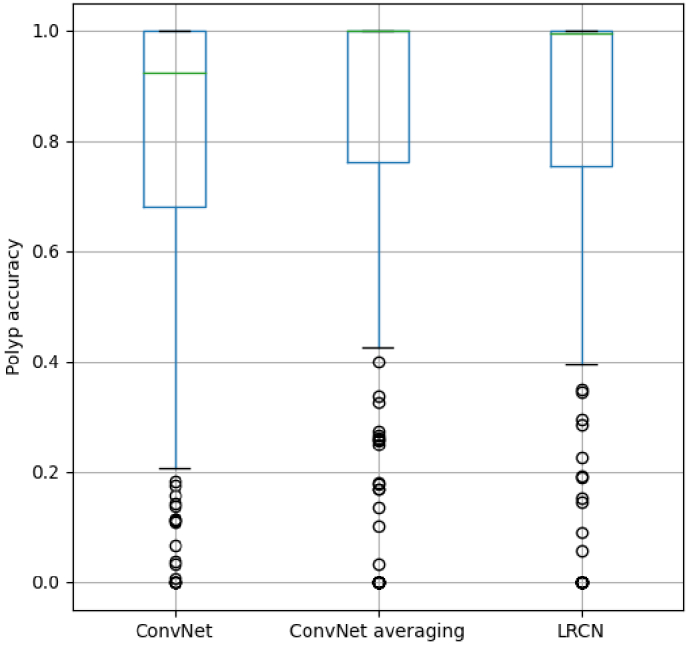
Boxplots showing the per-polyp accuracies for each method.

The per-polyp accuracy also increased for all temporal methods, with a higher improvement on the LRCN, showing that most polyps benefit from the temporal information, rather than just a few longer polyp sequences. Additionally, Fig. 4 shows the results from these experiments with a focus on the results per polyp. Boxplots are presented for the per-polyp accuracy, where the accuracy for each polyp is computed as the ratio of correctly classified samples in a polyp. It can be observed that both temporal methods improved the per-polyp results. The median of polyps accuracy increased to nearly 100%, and the fourth quartile increased 20% for both models. Polyps that had a high accuracy improved with temporal methods. Contrary, polyps that presented low accuracies (<50%) with the baseline presented an even lower accuracy with the temporal methods. Therefore, low polyp-accuracy outliers remain in both cases. Overall, the use of these techniques increased the consistency of the predictions within the same polyp, which can be further observed in the example timelines presented in Fig. 3. For the same polyp, the baseline ConvNet predicted most frames correctly, but yielded a considerable amount of mispredictions, with low temporal coherence. Both temporal methods increased the consistency of the predictions, the LRCN in this lesion yielding a 100% accuracy.

### 3.4 Comparison between temporal methods

In order to gain some understanding of the benefits of the different temporal methods, further analysis was performed. The temporal methods were evaluated in terms of the amount of temporal information present in the clips. The similarity of the frames within a clip was quantified by the means of normalised cross-correlation (Eq. (4)). The normalised cross-correlation (NCC) between consecutive frames was computed and averaged across each clip. High NCC values indicate small appearance variations within a clip.

(4) $$R(x,y)=\frac{\sum_{x\prime ,y\prime }(I_{i+1}^{\prime }(x\prime ,y\prime )\cdot I\prime (x+x\prime ,y+y\prime ))}{\sqrt{\sum_{x\prime ,y\prime }I_{i+1}^{\prime }{\left(x\prime ,y\prime \right)}^{2}\cdot \sum_{x\prime ,y\prime }I\prime {\left(x+x\prime ,y+y\prime \right)}^{2}}}$$

where I denotes an image, $I_{i+1}$ the following image and R the NCC result. The summation is done over the image pixels: x′=0⋯w−1 , y′=0⋯h−1 (where w and h are the width and height of the frame).

**Fig. 5.**
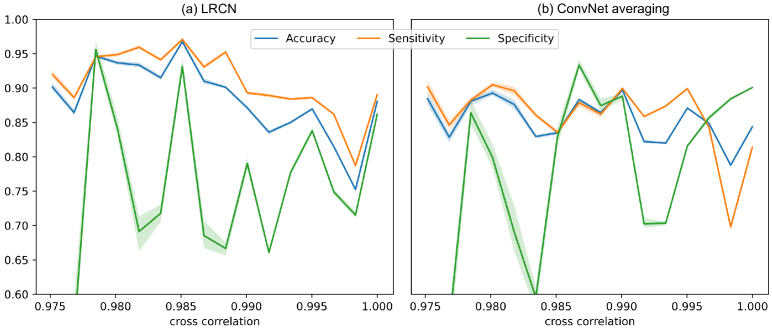
Performance for (a) LRCN and (b) ConvNet averaging for different clip cross-correlations - higher cross-correlation implies higher intra-clip similarity and lower variation. 95% confidence intervals are shown with transparency.

Figure 5 shows how the performance varies for different clip similarities. A Pearson’s correlation statistical analysis with α=0.05 was performed. For LRCN, it was observed that all performance metrics were negatively correlated to the similarity of a clip, but only the accuracy and sensitivity reached the critical value for statistical significance. Contrary, the ConvNet averaging method only showed a statistically significant decrease in sensitivity but not accuracy or specificity. Overall, the results suggest that the performance of LRCN decreases when clips show a high cross-correlation and that the ConvNet averaging method is not importantly affected by the amount of new information within the clip. In both cases, the specificity is unstable, possibly because the negative non-adenoma class is under-represented in our dataset.

**Fig. 6.**
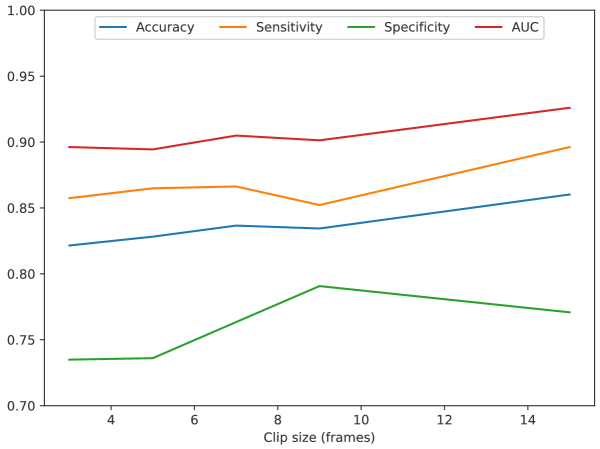
LRCN performance when trained with different clip sizes.

To further assess if the LRCN benefits from increased temporal information, the model was trained and evaluated with different clip lengths ranging from 3 to 15-frame clips. It is important to note that increasing the clip size considerably reduced the number of available clips. Figure 6 shows that the performance tended to improve with longer clips, with gains in accuracy and AUC, showing that LRCN may benefit from longer clips integrating higher temporal variation.

**Fig. 7.**
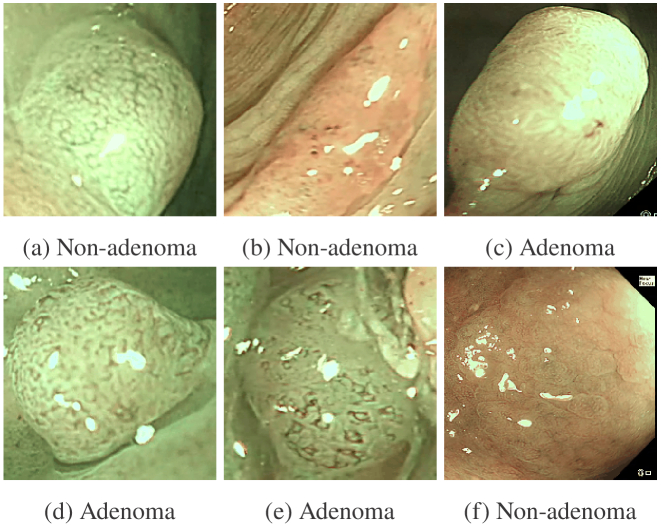
Classification results examples. The top row shows examples where ConvNet averaging succeeds and LRCN fails, and the bottom row examples where the opposite occurs.

Example clips are shown in Visualization 1. Figure 7 shows some example results from LRCN and ConvNet averaging. The top row shows frames from 15-frame clips where ConvNet averaging correctly classified but LRCN failed, and the second row vice-versa. ConvNet averaging performs better when classifying non-adenomas as it has a higher specificity, whereas the LRCN succeeds more at the classification of adenomas.

### 3.5 Detection and classification pipeline

The proposed methods for polyp type classification use the polyp region in the image, defined by a bounding box, as an input to the models (the area inside the blue boxes depicted in Fig. 1). All previously presented experiments use the expert annotations for the location of the polyp to train and test the networks. Nevertheless, it is important to evaluate the methods in a realistic setup where the polyp location is unknown. An additional experiment was therefore performed using a polyp detection model to obtain the polyp bounding box location in each image prior to the polyp classification methods, simulating the real workflow in a clinical setup. Regarding the performance of the polyp detection network, a total of 353 images were discarded due to polyp detection false negatives (98.72% sensitivity). Additionally, the detection network yielded 2,413 false positives (91.88% precision), generating less accurate boxes containing part of the background mucosa.

The results using the detection model are presented in Table 3. When compared to the results presented in Table 2, all diagnosis methods show a drop in performance, possibly due to a lower quality of the polyp localisation from partial views of the polyp. However, the temporal methods show a lower drop in polyp accuracy than the ConvNet baseline, showing that the overall improvement in predictions consistency is maintained even when predicted boxes can be temporally unstable. Particularly, the specificity of ConvNet averaging improved when using predicted boxes regions, bringing an overall small increase in accuracy.

**Table 3.** Polyp diagnosis cross-validation results on the internal dataset using predicted polyp boxes.

Method	Clip size	Accuracy(%)	Sensitivity(%)	Specificity(%)	AUC (%)	Polyp accuracy(%) [95% CI]
ConvNet	N/A	78.89	79.30	77.89	86.91	73.49 [69.17, 77.80]
LRCN	15	84.51	**88.73**	73.71	90.45	**79.21 [74.49, 83.93]**
ConvNet averaging	15	**84.69**	84.35	**85.56**	**91.16**	78.17 [73.41, 82.93]

**Fig. 8.**
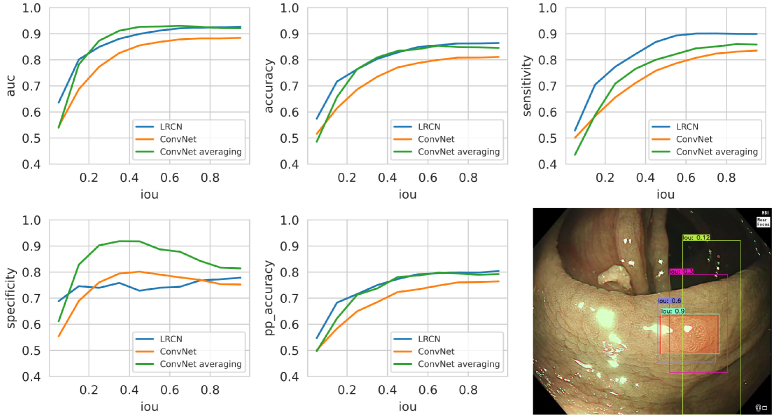
Models performance based on the quality of the position of the polyp box. The bounding box around each polyp was randomly moved to achieve 9 new boxes with an intersection over union (iou) with the original expert box ranging from 0.05 to 0.95. Area under the curve (auc), accuracy, sensitivity, specificity and per-polyp accuracy are shown. The image on the bottom right shows an example of the position of the original box (red transparency) and boxes obtained with different ious.

An additional experiment was carried out to evaluate the effect of the quality of the polyp box, the hypothesis being that the performance of the classification model is correlated with the quality of the crop. Each polyp was therefore evaluated using boxes with varying ranges of intersection over union (IoU) with respect to the original box. Each polyp in each frame was evaluated 9 times using boxes randomly generated presenting IoUs going from 5% to 95% with a 5% jump, so that all the IoU range was evaluated for each case. The image in the bottom right of Fig. 8 shows a few examples of random boxes with different IoUs for the same polyp.

The graphs in Fig. 8 show the results of the polyp diagnosis models when using the generated random boxes to extract the polyp region. The results are presented as a function of the IoU. As it can be observed, for all metrics the results improve for higher IoU values, showing that the quality of the polyp detection is important for diagnosis purposes. However, the performance plateaus when the IoU reaches approximately 50%, indicating that the classification performance is robust to minor discrepancies in the position of the polyp box. For most metrics the temporal methods reach a better performance than the baseline at lower IoU values (from an IoU of 0.2), showing that they could be used more reliably when the polyp detection boxes have lower quality. Interestingly, the specificity curves behave differently from the remaining performance metrics, showing a peak for the Convnet based methods at approximately IoU=0.4 and the LRCN presenting a more linear increase rather than exponential. This difference could be due to the fact that non-adenomas can present more similarities to normal mucosa than adenomas, and intermediate intersection over unions (0.3 - 0.7) would contain a partial portion of the polyp as well as some background. The ConvNet performance is more skewed towards higher specificity with the default threshold ( T=0.5 ) than for the LRCN, which could explain the bump on specificity when healthy mucosa is present in the box.

### 3.6 External dataset evaluation

The performance was measured on the publicly available dataset [26] described in Section 2.1. To test on this dataset, each of the 5-fold models was used in an ensemble to generate the final results for each method, using arithmetic mean combination. Table 4 shows the results for the ConvNet baseline and the temporal methods. Results on the Piccolo dataset were found to be comparable to the results on our internal data, with a drop of approximately 3% in accuracy for all methods when compared to the results in Table 2. The sensitivity in this dataset was lower, but a higher specificity was obtained, as well as slightly improved polyp accuracies. The number of polyps on this dataset was limited, especially due to the fact that some polyps were excluded from the evaluation as they did not contain 15 frames. The low number of samples was reflected in the large confidence intervals obtained for the polyp accuracy results.

**Table 4.** Polyp diagnosis ensemble results on the Piccolo Dataset.

Method	Clip size	Accuracy(%)	Sensitivity(%)	Specificity(%)	AUC (%)	Polyp accuracy(%) [95% CI]
ConvNet	N/A	77.36	72.83	98.02	96.81	78.08 [69.06, 87.09]
LRCN	15	**83.05**	**78.95**	**100.00**	97.60	**82.20 [70.72, 93.68]**
ConvNet averaging	15	82.20	77.89	**100.00**	**100.00**	77.88 [62.27, 93.50]

Both temporal methods improved the per-frame performance, showing a higher AUC. Particularly, both temporal methods show a 100% specificity in this dataset. LRCN showed an overall higher polyp accuracy, approximately 4% above the baseline, showing higher generalisability than the averaging method for the set threshold of 0.5.

## 4. Discussion and conclusion

In this paper, two approaches to exploit spatio-temporal features for polyp diagnosis were investigated. CADx systems for polyp diagnosis have shown promising results in previous literature. However, one of the limitations of these models is the inconsistency of predictions on the same polyp. To tackle this problem, we implemented two methods to incorporate temporal information in the predictions and improve the performance and the overall polyp accuracy.

First, we showed that implementing a simple temporal averaging over consecutive frames increased the performance of a CADx system and considerably improved robustness when applied to video data. Similarly, a more complex temporal model, LRCN, also yielded an improvement in performance and robustness. Although both methods were found to have comparable performance, our results indicated that the LRCN approach may benefit from larger temporal variations within the window, which potentially indicates a favorable performance of this method on longer videos.

Both of the proposed methods were evaluated on internal data and on an openly available external testing set. Cross-validation was used on our internal dataset, to ensure more representative evaluation results. The methods were additionally compared to a spatial baseline, providing ablation studies for fair comparison. The performance on the open dataset was found to be comparable to the results on our internal data, supporting the fact that temporal information brings an increase in polyp diagnosis performance. Furthermore, the fact that the external dataset did not contain consecutive frames from each polyps confirmed the generalisation capabilities of the temporal implementations. It is important to point out that large-scale colonoscopy polyp diagnosis datasets are lacking for both training and evaluation, but the problem is gaining traction, for instance in the GIANA Endovis challenge [42].

Such a CADx model would be used in combination with a polyp detection model, where the workflow would be to first detect a polyp in the image, and then use the detected polyp region to obtain a polyp classification between adenoma and non-adenoma. As this study aims to solve problems that arise from clinical use of such a device, it is imperative to test the full setup to provide realistic results. A polyp detection model was therefore implemented and run on the test videos. The obtained boxes were used for the different polyp diagnosis methods. The results showed that the effect of using non-expert boxes was minimised when using temporal diagnosis methods. An additional experiment evaluated the performance of each of the classification techniques based on the quality of the polyp boxes. This analysis demonstrated that the diagnosis capabilities were enhanced when the quality of the boxes improved, providing a practical clue for its use in a clinical environment, where clinicians could discard diagnosis predictions if the boxes are visually unsatisfactory. The results also show there might be scope to improve the classification performance on low-quality boxes through the use of more extreme data augmentation techniques. It is unclear where the turning point is where the position of a box becomes inaccurate enough that it hides important features needed for polyp classification.

Future work includes the use of other spatio-temporal techniques, as well as the inclusion of spatio-temporal data augmentation to decrease overfitting with small datasets. It was observed that the information present in a clip could affect the performance of a spatio-temporal model. This leads to think that there could be room to optimize the frames to use in a clip in a way that the information present is maximised. In this sense, the inclusion of sampling techniques should be explored as future work.

## Data Availability

Data underlying the results presented in this paper are not publicly available at this time due to permissions in ethics collection. Data from the External Dataset are available in Ref. [26].
